# Degradability Enhancement of Poly(Lactic Acid) by Stearate-Zn_3_Al LDH Nanolayers

**DOI:** 10.3390/ijms13077938

**Published:** 2012-06-26

**Authors:** Mahboobeh Eili, Kamyar Shameli, Nor Azowa Ibrahim, Wan Md Zin Wan Yunus

**Affiliations:** 1 Department of Chemistry, Faculty of Science, Universiti Putra Malaysia, Serdang, Selangor 43400, Malaysia; E-Mail: kamyarshameli@gmail.com; 2 Department of Chemistry, Centre for Defence Foundation Studies, National Defence University of Malaysia, Kuala Lumpur 57000, Malaysia; E-Mail: wanmdzin@upnm.edu.my

**Keywords:** poly(lactic acid), nanocomposites, layered double hydroxide, biodegradability enhancement, flexibility improvement

## Abstract

Recent environmental problems and societal concerns associated with the disposal of petroleum based plastics throughout the world have triggered renewed efforts to develop new biodegradable products compatible with our environment. This article describes the preparation, characterization and biodegradation study of poly(lactic acid)/layered double hydroxide (PLA/LDH) nanocomposites from PLA and stearate-Zn_3_Al LDH. A solution casting method was used to prepare PLA/stearate-Zn_3_Al LDH nanocomposites. The anionic clay Zn_3_Al LDH was firstly prepared by co-precipitation method from a nitrate salt solution at pH 7.0 and then modified by stearate anions through an ion exchange reaction. This modification increased the basal spacing of the synthetic clay from 8.83 Å to 40.10 Å. The morphology and properties of the prepared PLA/stearate-Zn_3_Al LDH nanocomposites were studied by X-ray diffraction (XRD), transmission electron microscope (TEM), scanning electron microscope (SEM), thermogravimetric analysis (TGA), tensile tests as well as biodegradation studies. From the XRD analysis and TEM observation, the stearate-Zn_3_Al LDH lost its ordered stacking-structure and was greatly exfoliated in the PLA matrix. Tensile test results of PLA/stearate-Zn_3_Al LDH nanocomposites showed that the presence of around 1.0–3.0 wt % of the stearate-Zn_3_Al LDH in the PLA drastically improved its elongation at break. The biodegradation studies demonstrated a significant biodegradation rate improvement of PLA in the presence of stearate-Zn_3_Al LDH nanolayers. This effect can be caused by the catalytic role of the stearate groups in the biodegradation mechanism leading to much faster disintegration of nanocomposites than pure PLA.

## 1. Introduction

Nowadays a great amount of various petroleum based polymers such as polyolefins, poly(vinyl chloride) and polystyrene are used. It is estimated that their production exceeds 10^8^ t/a [1]. Since these polymers are highly stable towards most environmental conditions, their consumption and discarding into the environment are major contributions to non-degradable wastes. Although recycling is an environmentally attractive solution, only a minor portion of plastics is recycled, and most of these wastes end up in municipal burial sites [2]. In addition, satisfactory landfill sites are also limited and disposal of these wastes in incineration leads to the production of toxic or noxious products that contribute to global pollution. Therefore, it seems that the best solution to overcome these problems is the development of biodegradable polymers or green polymeric materials [3,4].

Biodegradable polymers are referred to as those environment friendly polymers that can be enzymatically or hydrolytically degraded after a limited time of exposure to humidity, light or microorganism without releasing any toxic or noxious gases [5].

Factors such as the chemical structure and the molar mass of the polymers, the rigidity of the polymer chain, the removal and dissolution of fission products from the surface, the surface activation of the enzyme, the kind and value of the microorganisms existing in the environment, *etc*. are determining factors to control the degradation process [6,7].

Depending upon the nature of the biodegradable polymers, their degradation might take three to twelve months [8]. Different biological, chemical and physical phenomena can be responsible for the biodegradation of polymers. In the environment (soil, compost, municipal waste deposits, *etc.*), polymers are usually degraded by microbial consortia. The biodegradation level can be monitored by determining the carbon dioxide evolution derived from the test samples or oxygen consumption by them. However, the most common biodegradability test is the soil burial test. In this method, biodegradability is studied by evaluating the weight loss of the samples over a specific time in a soil environment [9].

Aliphatic polyesters are a main category of biodegradable polymers. The degradation of aliphatic polyesters is a complex process, which consists of mainly four phenomena: water absorption, ester cleavage and formation of oligomer fragments, diffusion of soluble oligomers and solubilization of fragments [10]. Poly(lactic acid) (PLA, from L-Lactic acid, D-Lactic acid or mixes of both) is one of the most popular members of this group that can be naturally degraded in soil or compost by fungi (*Aspergillus niger* and *Aspergillus flavus*) or by enzymes [6]. Although PLA presents a much slower soil degradation rate compared to the other aliphatic polyesters like polybutylene succinate (PBS), polybutylene adipate (PBA) and poly(ɛ-caprolactone) (PCl), it can be completely degraded into water and carbon dioxide without any eco-toxicological effect [11–13].

The biodegradability, thermoplasticity, transparency and fabricability of PLA as well as its high strength and high modulus make it a promising material for tissue engineering, service wares, waste-composting bags, mulch films, controlled release matrices for fertilizers, pesticides and herbicides [14,15]. However, its applications are limited due to its brittleness and nonflexibility, high crystallinity, slow degradation and costliness [16].

The effect of the montmorillonite (MMT) silicate layers on the biodegradability of aliphatic polyesters has been an interesting aspect of the research in the field of polymer/clay nanocomposites [17]. The research on the biodegradability of the PCL/MMT and PLA/MMT nanocomposites revealed a significant improvement of the biodegradability of the neat PCL and PLA after nanocomposite preparation with organo-modified MMT. However there is not any report in the literature regarding the effect of layered double hydroxide (LDH) nanolayers on the biodegradability of aliphatic polyesters.

LDHs or hydrotalcite-like compounds (HTs) are anionic clays which can be represented as [M(II)_1−_*_X_* M(III)*_X_* (OH)_2_]*^X^*^+^ [*A_X_*_/_*_n_^n^*^−^□*m*H_2_O]*^X^*^−^ where M (II) and M (III) are divalent and trivalent cations, respectively, and *A^n^*^−^ is an exchangeable anion [18].

LDHs are composed of octahedral M^2+^(OH)_6_ brucite-like layers which are positively charged by the partial substitution of M^3+^ for M^2+^. Thus, anions are intercalated into the interlayers to achieve charge neutrality [19]. The most useful property of LDH is its anion exchangeable ability that has made it attractive as a subject of many researches in different fields such as catalysts [20], ion exchangers [21], sorbents [22], electrochemistry [23], *etc*.

Anions are typically intercalated into LDH interlayers by three approaches [24]. The first approach is the co-precipitation method, which requires an addition of a solution of M^2+^ and M^3+^ ions into a base solution of the desired anions. The second technique is the direct ion exchange method, in which LDHs are stirred in a solution of the chosen anions at a suitable concentration. The last method is the rehydration method in which calcined LDH is added to a solution of desired anions [25]. The selection of anions for modification of LDH depends on the next application of LDH. For example, Mg–Al LDHs intercalated with dodecylsulfate and dodecylbenzenesulfonate have been used to adsorb pesticides, such as triadimefon, linuron, atrazine, acephate, and diazinon, from aqueous solution [26]. Also LDHs containing interlayer carboxylate anions have attracted considerable attention in recent years due to interesting properties and potential applications e.g., LDH modified with citrate, malate and tartrate ions are able to take up hazardous organic materials and heavy metal ions from an aqueous solution [27].

The interest in using LDH as a filler for polymer matrixes is given by some advantages of LDH which are lacking in more common fillers from the smectite group mineral compounds such as montmorillonite (MMT). Some of these specific advantages of LDH are: its higher ion exchange capacity, its highly tunable properties like particle size and aspect ratio, and its positively layered charge that can be modified with anionic surfactants [28]. The preparation and characterization of different polymer/LDH nanocomposites have been reported [29–36]. However, there are no reports on biodegradation studies of polymer/LDH nanocomposites. The objective of our research is to investigate the influence of the stearate-Zn_3_Al LDH nanolayers on the tensile properties and the biodegradability of PLA.

## 2. Results and Discussion

**Figure 1:**
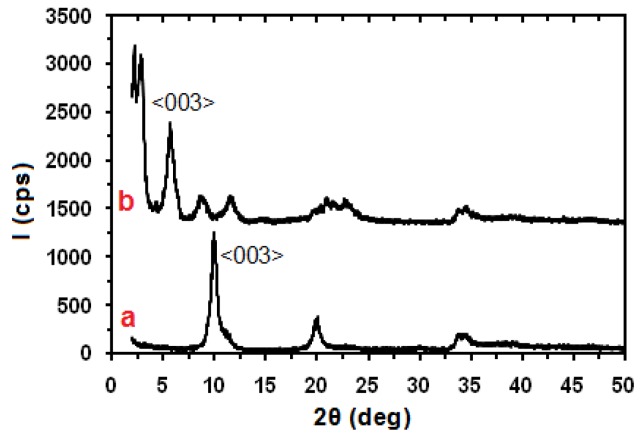
X-ray diffraction (XRD) pattern of (**a**) pristine Zn_3_Al layered double hydroxide (LDH) and (**b**) stearate-Zn_3_Al LDH.

The X-ray diffraction (XRD) patterns in the range of 2*θ* from 2 to 50° for the pristine and modified Zn_3_Al LDH are shown in Figure 1. It is apparent that both Zn_3_Al LDH and stearate-Zn_3_Al LDH are crystalline in nature with a well-defined layered structure. The basal spacing (*d*) of the Zn_3_Al LDH or stearate-Zn_3_Al LDH is calculated from the first diffraction peak using Bragg’s equation, *n*λ = 2 dsin*θ*, where n is equal to 1 for the <003> peak, *λ* is the wave length of Cu-Kα radiation, and *θ* is the half of the scattering angle. The modification of Zn_3_Al LDH with stearate ions increases the clay interlayer distance from 8.83 Å in Zn_3_Al LDH (corresponding 2*θ* value of <003> peak is 10.00°) to 40.1 Å in stearate-Zn_3_Al LDH (2*θ* = 2.156°). The increase of the basal spacing indicates that the anions are successfully intercalated into the interlayers of Zn_3_Al LDH [37].

**Figure 2:**
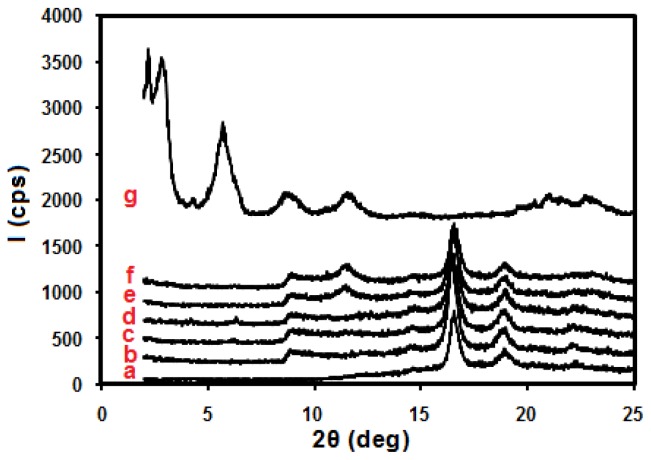
XRD spectra of (**a**) pure poly(lactic acid) (PLA) and the nanocomposites of PLA with (**b**) 1.0, (**c**) 3.0, (**d**) 5.0, (**e**) 7.0, (**f**) 10.0 wt % stearate-Zn_3_Al LDH content and (**g**) stearate/Zn_3_Al LDH.

X-ray diffraction patterns of the stearate-Zn_3_Al LDH, pure PLA and PLA/stearate-Zn_3_Al LDH nanocomposites containing various amounts of stearate-Zn_3_Al LDH are shown in Figure 2. As it can be observed, in the case of PLA/stearate-Zn_3_Al LDH nanocomposites, the diffraction peaks disappear indicating complete exfoliation of the LDH layers in the PLA matrix.

**Figure 3:**
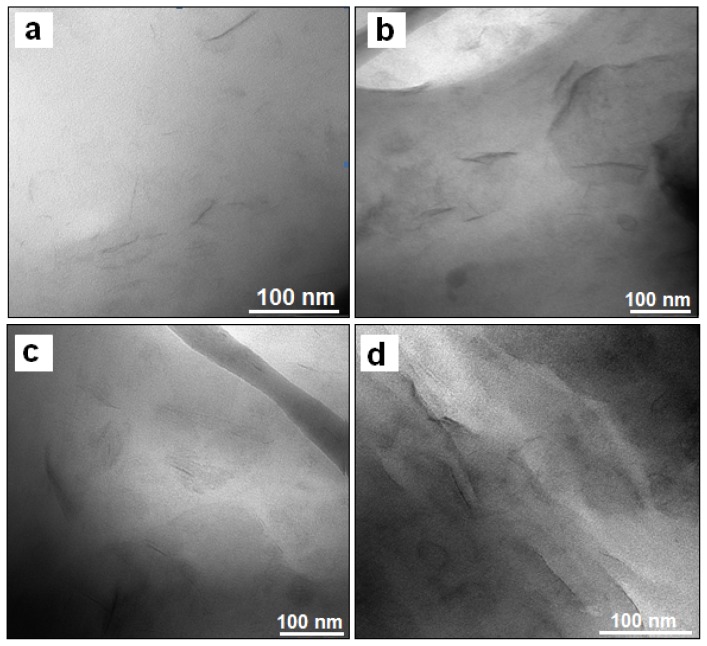
Transmission electron microscope (TEM) images of PLA/stearate-Zn_3_Al LDH nanocomposites with (**a**) 3.0, (**b**) 5.0, (**c**) 7.0, and (**d**) 10.0 wt % stearate-Zn_3_Al LDH content. (Magnification is 200 k).

The investigation of the internal structure of composites using XRD alone can often be misleading, since the XRD technique cannot detect layers that are in relatively disordered patterns in exfoliated nanocomposites [38] or it is unable to detect regular stacking that exceeds 88 Å in intercalated nanocomposites [39]. Figure 3 presents the high magnification transmission electron microscope (TEM) images of PLA/stearate-Zn_3_Al LDH nanocomposites containing 3.0, 5.0, 7.0 and 10.0 wt % stearate-Zn_3_Al LDH, respectively. The dark lines represent the LDH nanolayers, whereas the gray areas correspond to the PLA matrix. The images clearly reveal that the Zn_3_Al LDH layers have lost their stacking order and are randomly dispersed in the PLA matrix. The absence of aggregates confirms the high degree of exfoliation of the LDH plates within PLA matrix. Therefore the nanocomposites are the exfoliated type. The exfoliated structure observed here by TEM is in good agreement with the XRD results. Similar results are reported for Co Al LDH/polycaprolactone (PCL) nanocomposites [40].

**Figure 4:**
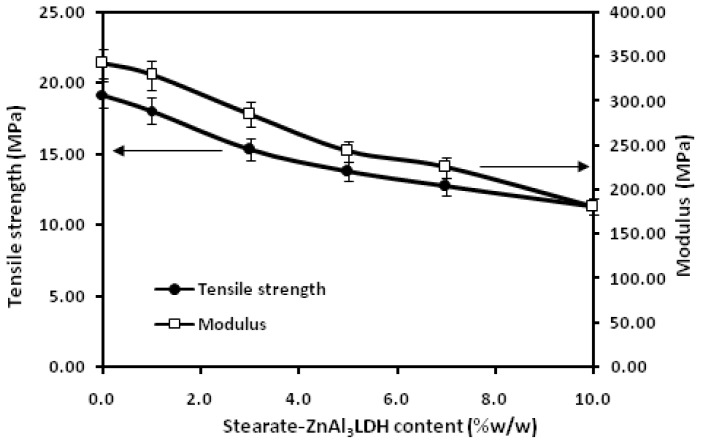
Tensile strength and elastic modulus of PLA with different stearate-Zn_3_Al LDH content.

**Figure 5:**
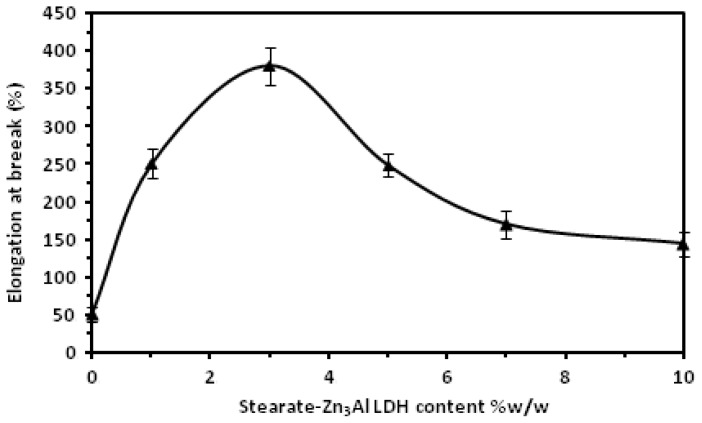
The elongation at break of PLA with different stearate-Zn_3_Al LDH content.

The dependences of the tensile strength, elastic modulus and elongation at break of the PLA/stearate-Zn_3_Al LDH nanocomposites on the stearate-Zn_3_Al LDH content are plotted in Figures 4 and 5. The error bars represent s.d. of at least two independent experiments in duplicate wells. Figure 4 shows that both tensile strength and elastic modulus decrease gradually as the filler content is increased, while Figure 5 displays that the addition of stearate-Zn_3_Al LDH content from 1.0 to 3.0 wt % into the PLA matrix leads to a dramatical increase (of more than 600%) of elongation at break. The diminution of tensile strength besides the flexibility improvement is usually reported for different polymers in the presence of various plasticizers [41–44]. It can be attributed to this fact that the plasticizer behaves like a solvent when mixed with a polymer, it causes the decrease of polymer chain cohesion and thus a reduction of tensile strength properties [45]. The significant increase in the elongation at break of the nanocomposites is maybe due to the presence of the long chain hydrocarbon parts of stearate anions in the modified stearate-Zn_3_Al LDH that act as a plasticizer. However the remarkable point is this that using a small amount of stearate-Zn_3_Al LDH promoted the flexibility and ductility of the final nanocomposites but did not decrease the tensile effectively, while the main drawback of most of the plasticizers that have been used for PLA matrix so far is the big drop of tensile strength and modulus of the products due to the large amount of plasticizer agent that is needed to plasticize it [46].

However, a further increase of the stearate-Zn_3_Al LDH content decreases the elongation at break. A high content of stearate-Zn_3_Al LDH can induce agglomerations of the filler that can cause reduction of elongation at break [47]. A similar finding was reported by Baiardo *et al.* [48] when acetyl tri-*n*-butyl citrate (ATBC) was applied as a plasticizer for PLA.

**Figure 6:**
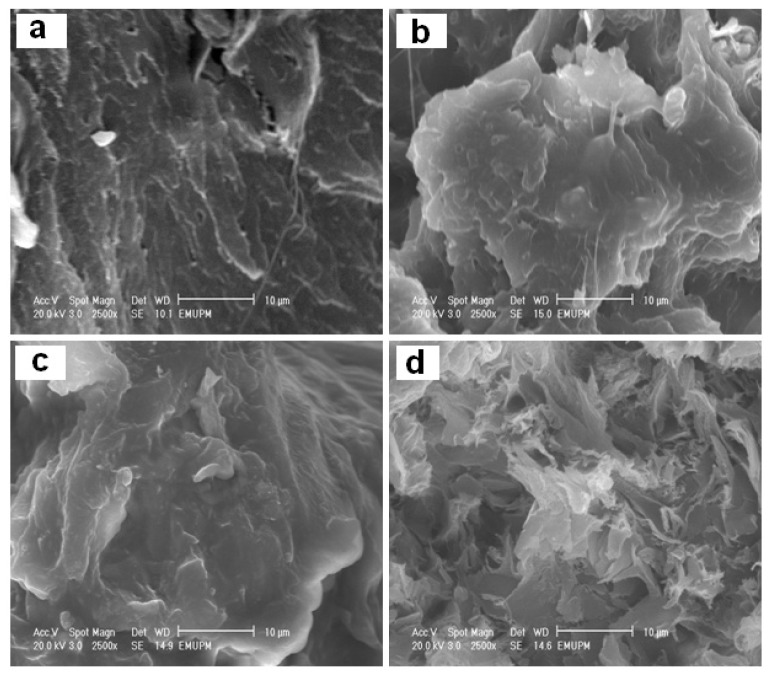
The fractured morphology of pure PLA (**a**) and PLA/stearate-Zn_3_Al LDH nanocomposites with (**b**) 1.0, (**c**) 3.0, and (**d**) 5.0 wt % stearate-Zn_3_Al LDH content. (Magnification is 2500 k).

Figure 6 shows scanning electron microscopy (SEM) micrographs obtained from the fractured cross-section surfaces of unfilled PLA and its stearate-Zn_3_Al LDH nanocomposites. The micrographs of unfilled PLA reveal rather compact solid and brittle fracture surfaces with little plastic deformation. Few long threads of a deformed material are observed on the fracture surface of PLA. On the entire fracture surfaces of the PLA with 1.0, 3.0 and 5.0 wt % of stearate-Zn_3_Al LDH, a large amount of plastically deformed material is discernible due to ductility and flexibility improvement of new nanocomposites. Similar images were obtained for PLA plasticized with poly ethylene glycol [41].

**Figure 7:**
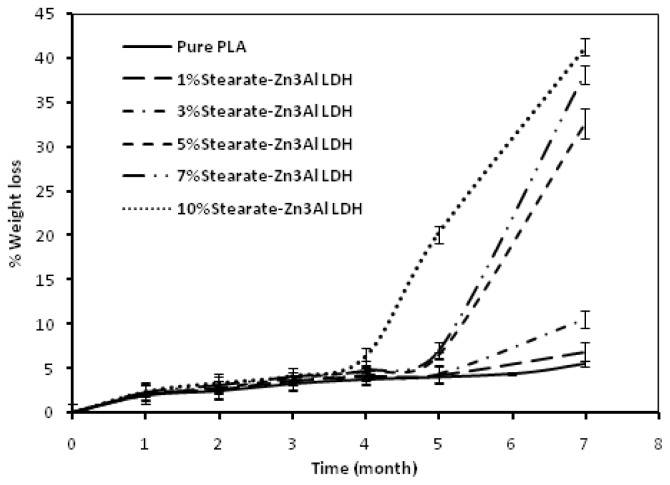
Weight losses of pure PLA and its stearate-Zn_3_Al LDH nanocomposites in soil for up to seven months.

Figure 7 shows the biodegradability of the pure PLA and PLA/stearate-Zn_3_Al LDH nanocomposites in soil for up to seven months. The nanocomposites exhibit a much higher disintegration rate than that of the pure PLA and the weight loss percentage increases as the filler loading is increased. The enhancement of biodegradation rate of PLA in soil as a result of the presence of stearate-Zn_3_Al LDH could be interpreted by the mechanism suggested by Ray *et al.* who found out that the disintegration rate of PLA is enhanced with the presence octadecyltrimethylammonium-modified MMT [49,50]. They suggested that this improvement might be caused by the existence of terminal hydroxylated edge groups in the silicate layers that homogeneously dispersed in the PLA matrix. These hydroxyl groups can absorb water from compost and start heterogeneous hydrolysis of the PLA matrix leading to faster degradation. The same mechanism can be assumed in the case of PLA/stearate-Zn_3_Al LDH nanocomposites, where the rates of weight loss of the PLA and PLA/stearate-Zn_3_Al LDH nanocomposites are almost the same up to around four months. However, after four months the weight loss rate of the nanocomposites is bigger than that of the unfilled PLA and a higher weight loss rate is observed when PLA/stearate-Zn_3_Al LDH is increased.

Another factor that may be associated with biodegradability improvement of the PLA nanocomposites is the faster degradation rate of stearate groups in the nanocomposite films than that of the PLA matrix. Therefore the PLA matrix will be broken down into smaller particles, resulting in an increased disintegration rate of nanocomposites films containing stearate-Zn_3_Al LDH than that of neat PLA film.

**Figure 8:**
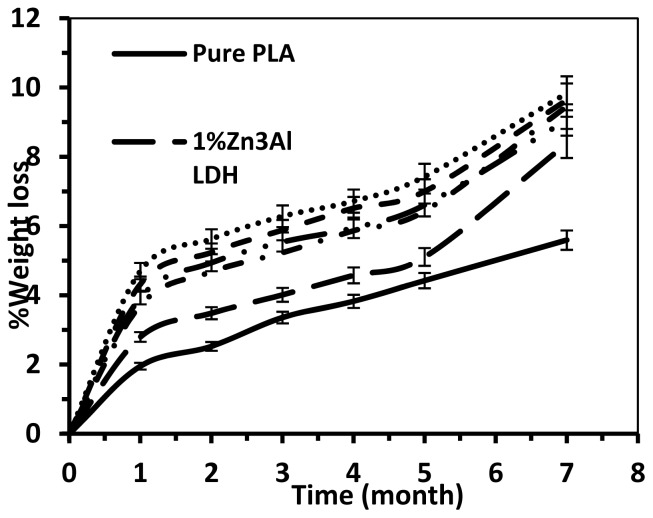
Weight losses of pure PLA and its Zn_3_Al LDH macrocomposites in soil for up to seven months.

The biodegradability of the pure PLA and PLA/Zn_3_Al LDH composites in the soil for up to seven months is shown in Figure 8. The rate of weight loss of macrocomposites is higher than that of the pure PLA. However, comparison of the results in Figures 7,8 shows that the degradation rate of nanocomposites is much higher than that of the macrocomposites. This may be due to the compactness of the unmodified LDH plates, which hinders water diffusion. These experiments were repeated three times for all of samples to show biodegradable properties.

Thermogravimetric analysis (TGA) measurements were carried out to obtain information on the thermal stability of the nanocomposite samples.

**Figure 9:**
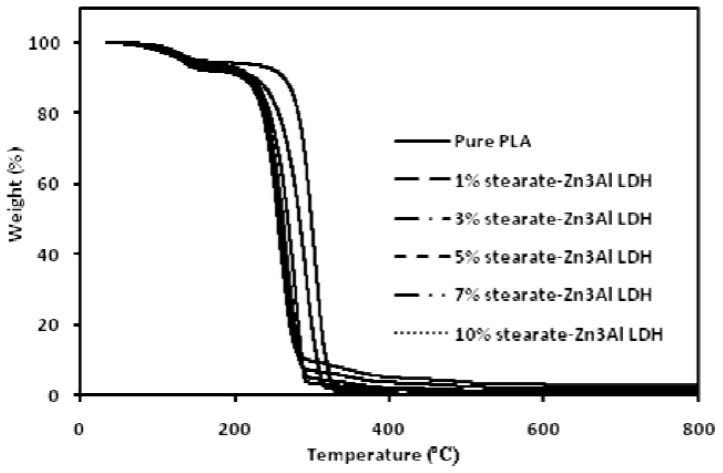
Thermogravimetric analysis (TGA) thermograms of the pure PLA and its stearate-Zn_3_Al LDH nanocomposites.

**Figure 10:**
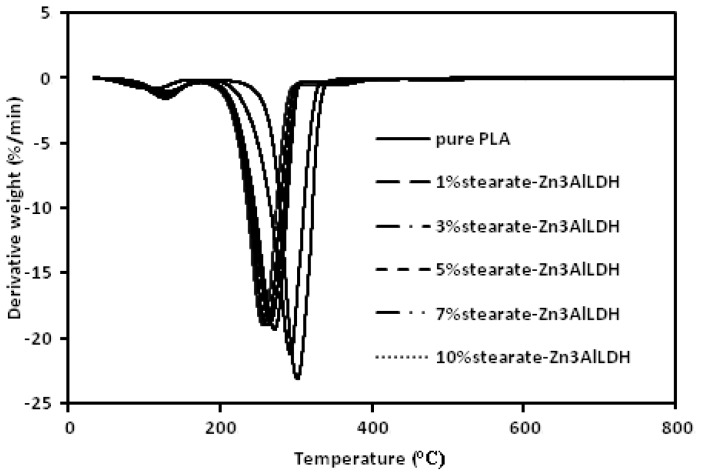
Differential thermal analysis (DTA) of the pure PLA and its stearate-Zn_3_Al LDH nanocomposites.

The TGA and differential thermal analysis (DTA) curves of the pure PLA and its stearate-Zn_3_Al LDH nanocomposites are presented in Figures 9 and 10, respectively. A two-stage decomposition process is observed for all samples, the first stage is maybe related to the loss of physically bonded water below 200 °C. However, the main decomposition step associated with the degradation of the PLA and its nanocomposites occurs at the temperature of 250–350 °C. It is clear that the *T*_onset_ of the PLA/stearate-Zn_3_Al LDH nanocomposites decreases as the amount of the stearate-Zn_3_Al LDH is increased. This reduction of *T*_onset_ may be attributed to the presence of long chain hydrocarbons, which may increase chain mobility and decrease strength and toughness. Since this product is useful for the packaging industry, this reduction cannot influence the next applications of products.

## 3. Experimental Section

### 3.1. Materials

Aluminium nitrate nonahydrate was supplied by HmbG (Germany). Zinc nitrate hexahydrate was purchased from Bendosen Laboratory Chemicals (Malaysia). Sodium stearate was purchased from R & M chemicals (UK). Sodium hydroxide pellets and chloroform were obtained from Merk (Germany) and Polylactide resin 4042D (92% L, 8% D) was supplied by NatureWorks LLC (USA). All the above-mentioned commercial chemicals were used as received.

### 3.2. Synthesis of Zn_3_Al LDH

The Zn_3_Al LDH was prepared by adding drop-wise a solution of NaOH (1 M) into a 250 mL solution of 22.30 g Zn(NO_3_)_2_·6H_2_O and 9.38 g Al(NO_3_)_3_·9H_2_O (with the mol ratio of 3 to 1) until pH 7 was obtained. The resulting suspension was shaken at 100 rpm and 70 °C for 16 h. The slurry was filtered, washed thoroughly with deionized water and dried at 60 °C for 24 h to obtain the Zn_3_Al LDH.

### 3.3. Preparation of Stearate-Zn_3_Al LDH

The stearate-Zn_3_Al LDH was prepared by replacing nitrate ions in the LDH layers with stearate ions using the following procedure. One gram of the dry Zn_3_Al LDH was first transferred into 750 mL of 0.003 M solution of sodium stearate solution and stirred at room temperature for 24 h. The white solid obtained was then filtered, washed with deionized water three times and dried in a vacuum desicator at room temperature.

### 3.4. Preparation of PLA/Stearate-Zn_3_Al LDH Nanocomposites

The nanocomposites of PLA with different amounts of stearate-Zn_3_Al LDH were prepared by a solution casting method. The desired amount of stearate-Zn_3_Al LDH in 40 mL chloroform was first sonicated for 3 h, transferred into a solution of 10 g PLA in 200 mL chloroform and refluxed for 24 h. The viscous suspensions were casted in a glass petri dish and dried in the solvent atmosphere to obtain the sample sheets.

### 3.5. Characterization Techniques

X-ray diffraction (XRD) patterns of the LDHs and composites were recorded using a Shidmadzu XRD 6000 Diffractometer at 30 kV and 30 mA with Cu-Kα radiation of the wavelength of 1.5405 nm.

Scanning electron microscopy (SEM) images were obtained using a Philips XL30 Environmental scanning electron microscope. The clean and dry samples were first coated with gold using a Bal-Tec SCD 005 sputter coater.

The transmission electron microscopy (TEM) images were obtained by employing a transmission electron microscope Hitachi, H7100 with an accelerating voltage of 200 kV. The samples were dispersed in chloroform and diluted to the right concentration. The suspension was then dropped on to the TEM sample grid and allowed to dry. The very thin layer on the grid was observed on the microscope.

The pure PLA and its nanocomposites were subjected to thermogravimetric analysis using Perkin-Elmer Thermal Analyzer (model TGA 7). The tests were carried out under a nitrogen gas atmosphere with a flow rate of 20 cm^3^·min^−1^ using a scan rate of 10 °C·min^−1^. The temperature range of 40 to 800 °C was used to study the thermal decomposition of the samples. The weight of each sample was about 30 mg. The weight loss of the samples during heating was automatically recorded and plotted as a function of temperature.

Tensile tests were performed by using a Universal Tester, Instron–4302, LEO. Tensile tests were performed according to the guidelines of ASTM D-638-V. The samples were cut into dumbbell shapes using a dumbbell cutter (Die BS 6476). The thicknesses of the samples were measured using a thickness gauge. A load cell of 1.0 kN was applied at a constant crosshead speed of 10 mm/min at room temperature. The reported results were the average of at least five measurements of tensile determinations (in duplicate).

Biodegradability of the samples was studied by measuring the weight loss of the thin-plate specimens buried in soil. Samples of 30 × 30 × 1 mm were weighed and then buried at a depth of 15 cm in the garden pots with an approximate capacity of 15 L and some holes at the bottom of the pots. The soil used in the tests was fertile soil for gardening without any pretreatment. The pots were placed in an uncovered gazebo with the temperature varying from 20 °C to 30 °C and a humidity above 80%. The buried samples were taken out once a month, washed in distilled water to remove the soil, dried in an oven at 60 °C for 24 h and the weight of dry samples was recorded. Based on the sample weight before and after degradation, the average percentage of weight loss for each sample was calculated. Weight loss of the specimens with time was used to indicate degradation rate in the soil burial test. The soil burial degradation test started on 5 October 2009 and ended on 5 May 2010.

## 4. Conclusions

Stearate-Zn_3_Al LDH, which was prepared by co-precipitation and ion exchange reaction, was used as nanofiller in a poly lactic acid (PLA) matrix. The internal structure of the nanocomposites in the nanometer range was established using X-ray diffraction and transmission electron microscope analysis. The effect of stearate-Zn_3_Al LDH loading on tensile properties and soil biodegradation was studied. Significant improvement of elongation at break was observed as a result of the addition of 1.0–3.0 wt % of stearate-Zn_3_Al LDH to the poly lactic acid. The presence of stearate-Zn_3_Al LDH nanolayers increased the rate of degradation of PLA in the soil. The thermogravimetric analysis showed that the presence of modified LDHs nanolayers does not lead to any improvement in thermal stability of PLA/stearate-Zn_3_Al LDH nanocomposites.
